# Diet quality and anxiety: a critical overview with focus on the gut microbiome

**DOI:** 10.3389/fnut.2024.1346483

**Published:** 2024-05-15

**Authors:** Melissa Basso, Irene Zorzan, Nicola Johnstone, Matteo Barberis, Kathrin Cohen Kadosh

**Affiliations:** ^1^School of Psychology, Faculty of Health and Medical Sciences, University of Surrey, Guildford, United Kingdom; ^2^Molecular Systems Biology, School of Biosciences, Faculty of Health and Medical Sciences, University of Surrey, Guildford, United Kingdom; ^3^Centre for Mathematical and Computational Biology, CMCB, University of Surrey, Guildford, United Kingdom; ^4^Synthetic Systems Biology and Nuclear Organization, Swammerdam Institute for Life Sciences, University of Amsterdam, Amsterdam, Netherlands

**Keywords:** diet, diet quality, anxiety, gut microbiome, sex stratification

## Abstract

Anxiety disorders disproportionally affect females and are frequently comorbid with eating disorders. With the emerging field of nutritional psychiatry, focus has been put on the impact of diet quality in anxiety pathophysiology and gut microbiome underlying mechanisms. While the relationship between diet and anxiety is bidirectional, improving dietary habits could better facilitate the actions of pharmacological and psychological therapies, or prevent their use. A better understanding of how gut bacteria mediate and moderate such relationship could further contribute to develop personalized programs and inform probiotics and prebiotics manufacturing. To date, studies that look simultaneously at diet, the gut microbiome, and anxiety are missing as only pairwise relationships among them have been investigated. Therefore, this study aims at summarizing and integrating the existing knowledge on the dietary effects on anxiety with focus on gut microbiome. Findings on the effects of diet on anxiety are critically summarized and reinterpreted in relation to findings on (i) the effects of diet on the gut microbiome composition, and (ii) the associations between the abundance of certain gut bacteria and anxiety. This novel interpretation suggests a theoretical model where the relationship between diet and anxiety is mediated and/or modulated by the gut microbiome through multiple mechanisms. In parallel, this study critically evaluates methodologies employed in the nutritional field to investigate the effects of diet on anxiety highlighting a lack of systematic operationalization and assessment strategies. Therefore, it ultimately proposes a novel evidence-based approach that can enhance studies validity, reliability, systematicity, and translation to clinical and community settings.

## 1 Introduction

Anxiety is one of the most prevalent mental health disorders, affecting up to 33.7% of the population with incidence in females twice as high as in men (1). With increasing global burden (2) – and an estimated 25.6% increase following the COVID-19 pandemic (3) – effective treatments and prevention programs are needed both at an individual- and community-level. Yet research has repeatedly shown that current frontline treatments, such as pharmacological interventions, have a limited efficacy for some patients (4) and that access to cognitive behavioral therapy remains limited.

With the emergence of nutritional and personalized psychiatry, research has uncovered the psychoactive potential of diet and paved the way for a more holistic approach to mental health (5). Our eating habits, which are deeply rooted in culture and geography, can vary widely. For instance, both the Mediterranean and Nordic diets emphasize healthful foods such as seasonal and whole foods, lean proteins, healthy fats, fruits, and vegetables. Similarly, vegetarian and vegan diets focus on plant-based foods yet do not distinguish between whole and processed options. A high intake of processed food rich in trans-fatty acids and added sugar, i.e., pre-packaged foods, refined grains, processed meat, sweetened drinks characterized the Western diet. Such dietary pattern has exponentially spread in the last 70 years and has been blamed for multiple non-communicable diseases, including mental health conditions (6). Nutritional research indicates that diets rich in antioxidants can reduce anxiety (7), while pro-inflammatory diets including high-sugar and high-processed food can heighten anxiety and cause neurochemical changes (8, 9). These effects could be mediated by the gut microbiome, which is strongly interconnected with both diet and anxiety. Indeed, studies showed that both dietary choices and gut microbiome influence anxiety symptomatology, and that healthy- and Western-like dietary patterns differentially affect gut bacteria (10–13).

Diet effects on anxiety symptomatology have already been systematically summarized elsewhere (10). The authors highlighted associations between lower (higher) level of anxiety symptoms or disorder prevalence and “healthy” (“unhealthy”) dietary patterns. In addition, they reported associations between a reduced anxiety and a higher intake of vegetables, fruits, micronutrients, omega-3 fatty acids, alpha-lipoic acid, omega-9 fatty acids, and associations between an increased anxiety and a higher intake of sugar and refined carbohydrates, and an inadequate intake of tryptophan and protein. However, they made no distinction between dietary effects on anxiety in females and males. The literature suggests that diet presents with sex-specific effects in relation to anxiety. For example, a positive association between anxiety and legumes consumption was found in women but not in men (14), a higher caffeine intake was associated with higher odds of anxiety in women but not in men (15).

In addition, Aucoin et al. (10) examined associations between anxiety and gut microbiome by focusing on gut microbiome targeting interventions only (probiotics, prebiotics, synbiotics). Additionally looking at gut microbiome alterations that have been specifically associated with anxiety could be helpful in pinpointing the underlying mechanisms. Furthermore, it could aid the development of personalized nutritional therapies by identifying next generation probiotics species to be tested in anxiety treatment – analogously to what is currently being done for major depressive disorder (Zorzan and Barberis, personal communication) – and by taking into account sex-based variations. Indeed, sex-specific associations also occur between gut microbiome and anxiety severity at the species level as pinpointed in a study by Ganci et al. (16): in males, *Alistipes shahii* was negatively associated with anxiety; in females (males), *Lactobacillus paracasei* (*Lactobacillus plantarum*) and *Streptococcus dysgalactiae* (*Streptococcus gallolyticus*) were positively associated with anxiety. Jiang et al. (17) also observed that probiotics supplementation differentially alleviates anxiety-like behaviors in male and female animal models. They found that females are more susceptible than males to gut dysbiosis and dysfunctions of the intestinal barrier and the blood–brain barrier. Altogether, the results suggest that a sex-focused approach may be adopted. Other than in-patient settings, such considerations could be most influential for prevention and in subclinical and community settings for those with first anxiety onset. Dietary interventions could then be used as a cost-effective and easily accessed tool to be used prior to pharmacological and psychological intervention, or alongside low-level therapies.

Finally, Aucoin et al. (10) did not evaluate specific diet assessment and operationalization strategies leading to a lack of interrogation of conflicting outcomes across studies. The authors also conflated human and animal studies, possibly leading to unjustified conclusions (18). Indeed, translational validity of preclinical studies has been questioned repeatedly and some authors have pointed out reliability, predictability and safety concerns that warrant a distinct level of cautiousness when interpreting results derived from animal models and human-testing methods (19, 20).

Based on the above, the current study will (i) critically examine the relationship between diet and anxiety while discussing the employed methodology, and (ii) reinterpret such findings in relation to gut microbiome, emphasizing translational opportunities. Section 2 will elaborate a theoretical model including connections between diet, gut microbiome, and anxiety. Section 3 will summarize the impact of diet on anxiety, assess methodologies for clarity on conflicting results, and suggest a new evidence-based approach. In parallel, it will also highlight key findings on diet’s influence on gut microbiome composition. Section 4 will outline associations between gut microbiome and anxiety and integrate pairwise relationship to identify bacterial genera that could mediate dietary effects on anxiety. Finally, this section will discuss the possible translation of such findings on interventional opportunities in the form of probiotics and prebiotics.

## 2 Connection between diet, gut microbiome, and anxiety: a theoretical model

**Figure 1:**
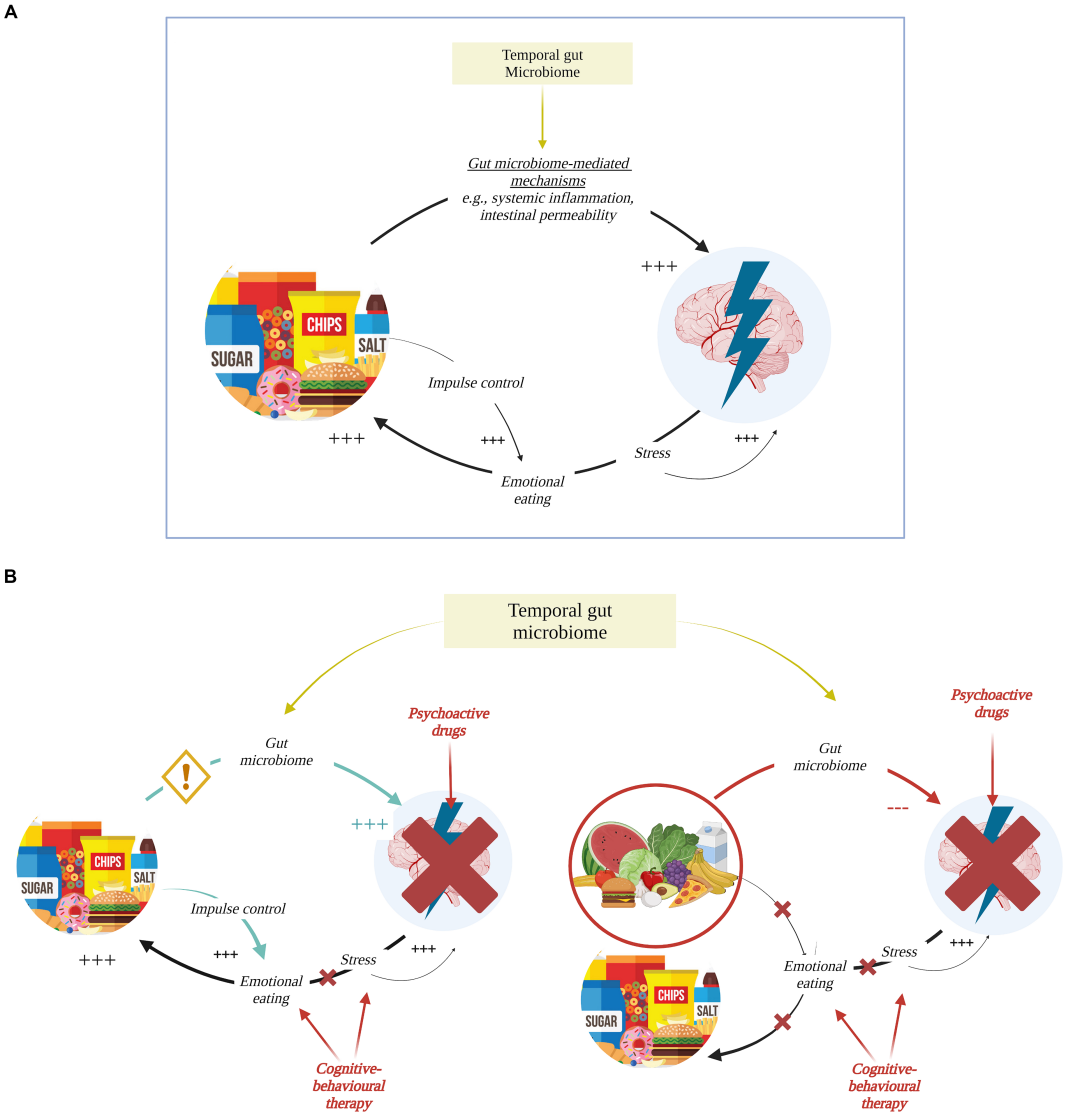
Bi-directional relationship for diet and anxiety. **(A)** Diet and anxiety feed each other in a vicious cycle: processed food increases (+++) anxiety through multiple gut microbiome-mediated mechanisms (see Figure 2 for more details) with individual-specific effects dependent on stable features of one’s microbiome, i.e., temporal microbiome. In turn, anxiety and stress trigger emotional eating behavior perpetuating intake of comfort food as in Western-like diet. Comfort food then acts on the brain as an immediate reward while affecting impulse control and positively reinforcing the cycle. **(B)** Left: psychopharmacological and cognitive-behavioral therapies have limited effect on anxiety. Their efficacy might be affected by unhealthy eating habits that – when not targeted through nutritional interventions and educational programs – keep triggering (+++) anxiety symptomatology and reinforcing emotional eating. Right: shifting toward a more nutritious and healthy diet tailored on individual’s gut microbiome composition would give the opportunity to break that cycle and would complement cognitive-behavioral therapies aimed at stress and emotional management as well as psychopharmacotherapy when strictly required. Created with and adapted from BioRender.com.

**Figure 2:**
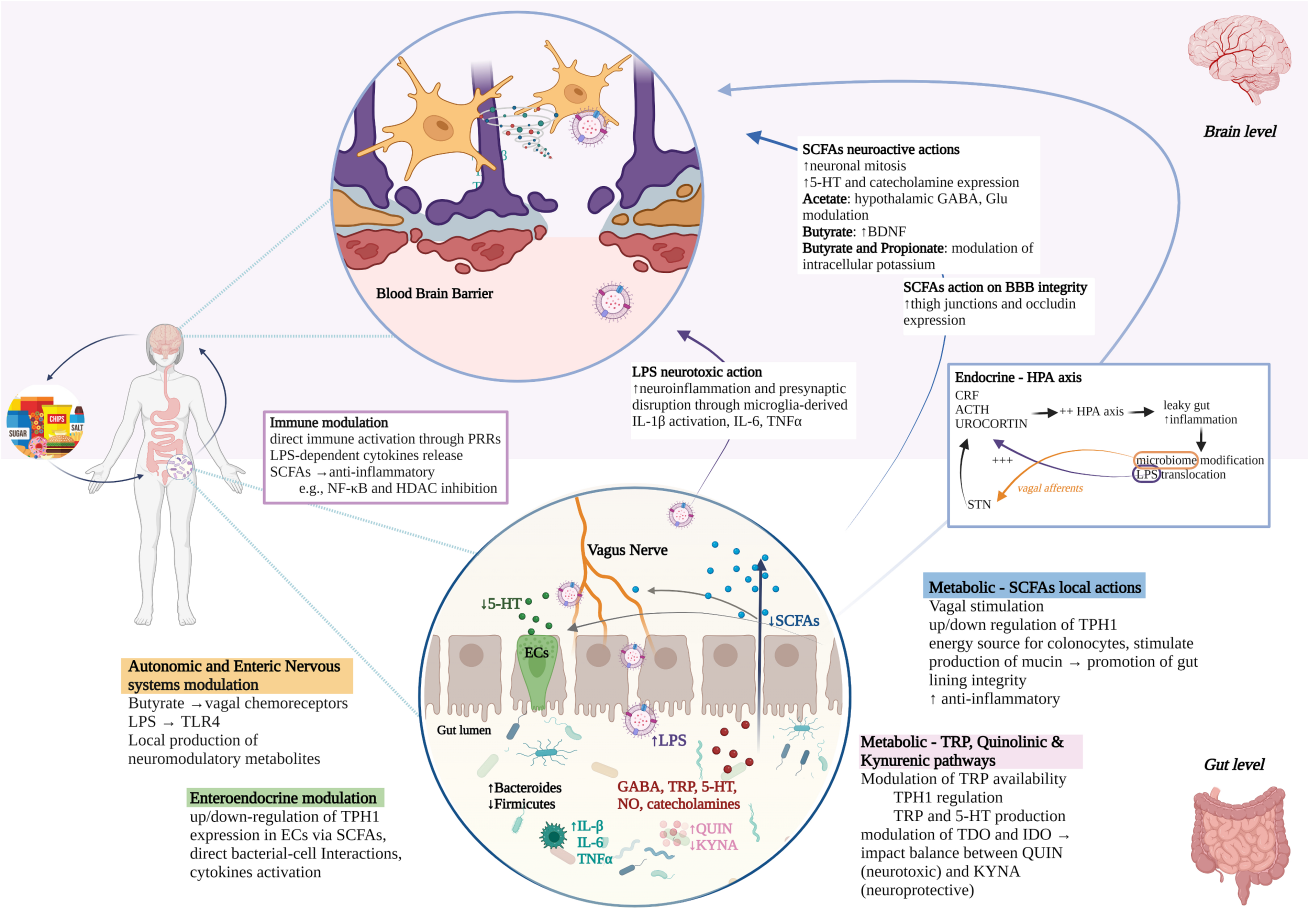
Gut microbiome-brain communication routes. Diet can act on mental health through gut-dependent mechanisms. Pathways of communication between the gut microbiome and the brain include processes happening in the gut (white background, bottom half), brain (pink background, upper half), and systemically (mid-figure), specifically: (1) autonomic and enteric nervous system modulation through, e.g., vagal chemoreceptors stimulation, TLR4-LPS binding, local production of neuromodulatory metabolites that can interact with enteroendocrine cells; (2) up/down-regulation of TPH1 expression in Ecs via direct and indirect mechanisms; (3) metabolic routes, i.e., (i) *SCFAs production* that can exert vagal stimulation, regulate TPH1 expression in Ecs, regulate the gut lining integrity and inhibit pro-inflammatory genes expression. SCFAs that enter circulation can also cross the blood–brain barrier (BBB) and promote neurogenesis, 5-HT, and other neurotransmitters expression other than increasing expression of BBB tight junction proteins such as occluding; (ii) modulation of TRP availability and TDO/IDO enzymatic activity in Quinolinic and Kynurenic pathways with consequent impact on balance between available TRP for 5-HT manufacturing, QUIN, and KYNA; (4) immune modulation through, e.g., direct activation through PRRs or indirect anti/pro-inflammatory actions of SCFAs and LPS respectively; (5) cyclic communication with the HPA axis where a molecular cascade leads to inflammation, leaky gut, and ultimately microbiome modifications and LPS translocation. LPS can in turn trigger stress-hormone release, whereas microbiome can modulate the HPA axis through the NTS. Stress, SCFAs production, inflammation, and gut microbiome disbalances interact together in promoting/disrupting gut lining integrity. In anxiety pathophysiology, highly processed diets could promote gut microbiome disbalances, e.g., increased *Bacteroides* and decreased Firmicutes (magnified gut circle), which in turn could be related to changes in production of neurometabolites (in red), decreased SCFAs (in blue) with consequent decrease of their protective functions, increased LPS (in purple) and LPS translocation with consequent neurotoxic and inflammatory actions, disruption of QIN/KYNA balance (in pink), increased inflammation (in turquoise) and leaky gut. Psychological stress and highly processed diets could then perpetuate the cycle and reinforce ongoing pathophysiological processes. TLR4, toll-like receptors; LPS, lipopolysaccharide; TPH1, tryptophan hydroxylase 1; Ecs, enteroendocrine cells; SCFAs, short-chain fatty acids; BBB, blood–brain barrier; 5-HT, serotonin; TDO, tryptophan 2,3-dioxygenase; IDO, indoleamine 2,3-dioxygenase; QUIN, quinolinic acid; KYNA, kynurenic acid; PRRs, patterns recognition receptors; HPA, hypothalamus-pituitary–adrenal; NTS, nucleus tractus solitarii; GABA, gamma-aminobutyric acid; NO, nitric oxide; TRP, tryptophan; IL, interleukin; CRF, corticotropin-releasing factor; ACTH, adrenocorticotropin hormone; BDNF, brain-derived neurotropic factor; TNFα, tumor necrosis factor; NF-κB, nuclear factor kappa B; HDAC, histone deacetylase. Created with and adapted from BioRender.com.

Defining the nature of dietary effects on anxiety is complex due to the existence of a cyclical feedback loop between emotions and behavioral responses that can be reinforced by the dietary content (see Figure 1A). On one hand, food components could act on brain and mental health via modulation of brain chemistry and support of brain structures. This could happen through gut-microbiome dependent mechanisms, including autonomic and enteric nervous system modulation, enteroendocrine and hypothalamic–pituitary–adrenal (HPA) axis signaling, production of metabolic bacterial by-products such as short-chain fatty acids (SCFAs), systemic and low-grade inflammation, damage of the intestinal mucosal barrier that in turn alters the gut microbial community (see Figure 2). As an example, some authors have reported prolonged consumption of sucrose and trans-fatty acids – players in low-grade chronic inflammation, gut permeability, and gut flora alterations – to increase anxiety in rats (21–23). On the other hand, anxiety promotes unhealthy habits through emotional eating behaviors, intended to soothe and suppress negative emotions and stress effects, which can be in turn boosted by food, e.g., sucrose via its decreasing effect on impulse control (8). Psychological stress could also act on the integrity of the intestinal and blood–brain barrier, facilitating bacteria by-products (e.g., lipopolysaccharide) translocation and altering the gut microbiome composition (24). Diet and anxiety can thus enter a maintenance feedback loop, while hindering the efficacy of existing treatments. It follows that it is essential to disrupt this cycle by adopting a multi-pronged approach that includes nutritional intervention and cognitive-behavioral therapies focused on enhancing impulse control and developing effective coping strategies (see Figure 1B).

Defining the role of the microbiome in this context could further enable customized dietary interventions based on individual gut microbial profiles. This approach could also identify specific bacterial strains and dietary choices that optimize the efficacy of psychobiotic interventions, namely probiotics and prebiotics (25). Preclinical research on mouse and rat models has showed mixed results as summarized in a review by Berding et al. (12). For example, a few studies reported dietary effects on gut microbiome but not anxiety-like behavior, and some studies reported anxiogenic effects of, e.g., the “Cafeteria diet” (i.e., Western-like dietary pattern used for animal models), and high-fat versus high-sucrose, and low-fat diets (26, 27). Along with behavioral effects, increased Firmicutes/Bacteroidetes ratio was found in both studies, whereas changes in Firmicutes families were found in a cafeteria diet study that showed no effects on anxiety-like behavior (28). In humans, only a little research has been done to concomitantly investigate diet, gut microbiome, and anxiety. For example, Johnstone et al. (29) found anxiolytic effects of galacto-oligosaccharides supplementation in anxious females, along with an increase of *Bifidobacterium* abundance. Similarly, Taylor et al. (30) reported an inverse relationship between anxiety scores and *Bifidobacterium* in females, but not in males, after adjusting for fiber intake. Despite the existence of dietary guidelines for improved health (31), the specific impact of diet on anxiety and its mechanisms via the gut microbiome remains uncertain, due to inconsistencies in research findings and gaps in the literature. Inconsistencies could stem from a variety of factors, primarily the complex physiological processes involved that have only recently begun to be addressed. For example, dynamic changes in microbiome composition driven by short-term dietary habits depend on one’s temporal microbiome, intended as stable features such as enterotypes, which in turn can be affected by long-term diet and prolonged psychological stress (32–35). The association between anxiety risk and gut microbes may also be influenced by enterotypes (35). Deleterious effects of unhealthy eating habits could then be exerted through immunomodulation and intestinal barrier disruption, which are in turn regulated by the gut microbiome (36). This complex interplay highlights the need to consider both moderating and mediating factors in research (12, 24, 32). Finally, the lack of consensus on diet operationalization strategies further contributes to heterogeneous results and challenges in study comparison.

## 3 Dietary effect on gut microbiome and anxiety: an evaluation of the available evidence

### 3.1 A diet quality approach

**Figure 3:**
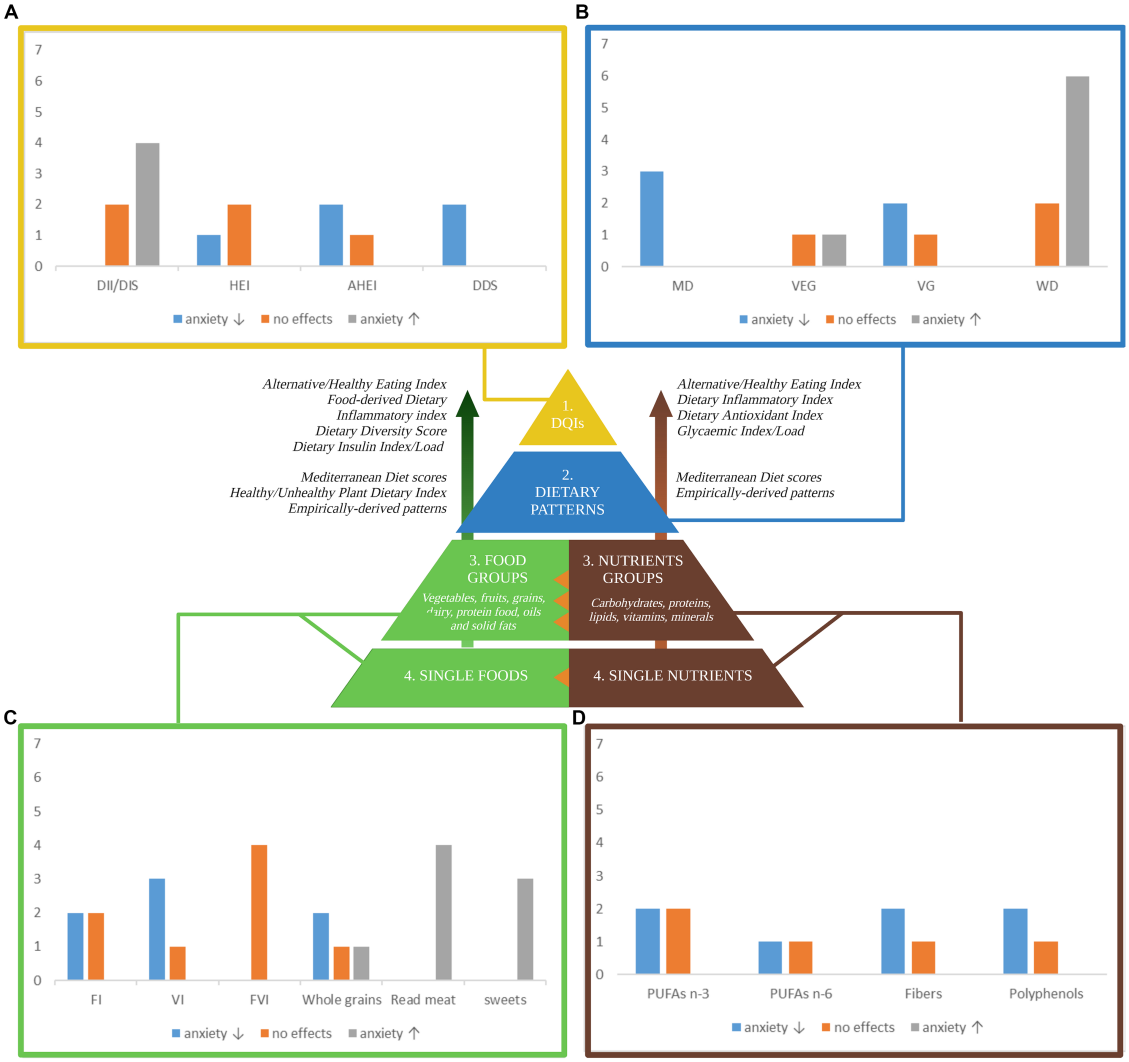
A pyramidal hierarchy of diet classification. Optimal and balanced nutrition is essential for good health. Human nutrition can be conceptualized in hierarchical levels: (1) overall diet quality as measured by several dietary quality indexes (DQIs) derived from distinct frameworks of reference including food and nutrient-derived ones, i.e., the Healthy Eating Index, based on the Dietary Guidelines for Americans, the Alternative HEI, adjusted for food associated to disease risk; nutrients-derived ones (right side) i.e. the Dietary Inflammatory Index assigning literature review-based inflammatory scores to 45 nutrients, the Dietary Antioxidant Index summarizing the total dietary antioxidant capacity, the Glycemic Index/Load indexing carbohydrates quality; food-derived ones (left side), i.e., the Food-derived Dietary Inflammatory Index, the Dietary Diversity Score quantifying diet variety and consumed unique food groups, the Dietary Insulin Index/Load quantifying the postprandial insulin response. (2) Dietary patterns as a complex combination of food and/or nutrients that can be either predefined or empirically derived. (3) Food groups and/or Nutrients groups are the units weighted to compute dietary patterns. (4) Single foods and single nutrients are grouped together to form food and nutrients group. The orange triangles highlight transversal hierarchical relationships: single nutrients synergically interact to form the food matrix conferring to foods their unique properties; nutrients groups are differently clustered across food groups. The bar charts in the four corners of the figure summarize the number of studies (y-axis) that investigate the associations between anxiety and **(A)** dietary quality indexes, **(B)** dietary patterns, **(C)** foods, and **(D)** nutrients. Sweets include sweet beverages; read meat includes processed meat. Blue, grey, and orange bars indicate that a higher index/score/consumption is associated with a decreased anxiety, an increased anxiety, and no effects on anxiety, respectively. Only the most relevant diet classification methods are included. DII, Dietary Inflammatory Index; HEI-2010, Healthy Eating Index-2010; AHEI-2010, Alternative Healthy Eating Index-2010; DDS, Dietary Diversity Score; MD, Mediterranean Diet; VEG, Vegetarian Diet; VG, Vegan Diet; WD, Western Diet; FI, fruits intake; VI, vegetables intake; FVI, Fruits and vegetables intake; PUFA n-3, omega-3 poly-unsaturated fatty-acids; PUFA n-6, omega-6 poly-unsaturated fatty-acids. Created with and adapted from BioRender.com.

A better understanding of how diet is linked to anxiety symptomatology is warranted for the development of targeted interventions. Up until now, nutritional standards have been conceptualized in terms of *quantity*, i.e., the right number of ingested calories and macronutrients based on demographics (37), lifestyle (e.g., physical activity level), and health (e.g., basal metabolism, chronic disease) factors. Recently, dietary recommendations have undergone a change toward a *quality*-centered approach as increasing evidence showed that food source and nutritional composition matters as much as numbers do. Diet quality refers to a “diversified, balanced, and healthy diet” (38) that supports good health and limits the risk of chronic disease through life by following specific food adequacy and moderation guidelines other than balancing energy intake and expenditure (31, 39). However, being focused on calories’ source, rather than calories alone, the evaluation of diet quality presents with several challenges due to (i) different frameworks of reference for “quality” definitions; (ii) the existence of multiple and hierarchical levels of diet conceptualization (see Figure 3); (iii) heterogeneous operationalization strategies within hierarchical levels. Hence, the nutritional research landscape is wide yet diverse and provides only sparse evidence of diet effects on anxiety that, if combined, could highlight overlaps and gaps in the literature while providing new insights and lay the foundation for further research into gut-microbiome mechanistic processes.

### 3.2 Diet quality indices

When conceptualizing diet hierarchically, diet quality indexes (DQIs) can be found on the top of the pyramid (see Figure 3). Following the need of *quantifying quality*, several DQIs have been created with similar yet distinct intents such as measuring pro-inflammatory diet potential, or alignment to existing guidelines. While all assess overall quality of dietary intake in a quantifiable and systematic manner, they can be either *food* or *nutrients*-derived, sometimes both, with considerable implications in results interpretation.

#### 3.2.1 Dietary inflammatory index and diet inflammation score

The Dietary Inflammatory Index (DII) is a literature-derived proxy for the diet inflammatory potential as expressed on a continuum from maximally anti-inflammatory to maximally pro-inflammatory. It reflects interval changes in inflammatory biomarkers such as C-reactive-protein (40). An alternative and recently developed measure of the contribution of diet to inflammation is the Dietary Inflammation Score (DIS). While the DII is based on 45 dietary components primarily including nutrients, the DIS relies on the intake of 19 food groups (41). Evidence exists for elevated inflammatory biomarkers in anxiety patients, and cytokines-mediated effects on anxiety related brain structures (e.g., heightened amygdala and insula activity, changes in functional connectivity between amygdala and prefrontal cortex). Also, chronic low-grade inflammation has been implicated in anxiety pathophysiology both in human and animal research and has been linked to the gut microbiome and a combination of lifestyle factors including diet (36, 42). Consistently, Zheng et al. (43) found that the diet inflammatory potential is associated with defined microbiome features, without appreciable sex-differences. In their study, pro-inflammatory diets were associated with increased abundance of certain Firmicutes species such as *Ruminococcus torques, Eubacterium nodatum, Acidaminococcus intestini, Clostridium leptum*; anti-inflammatory diets were associated with an increased abundance of the candidate probiotic species *Akkermansia muciniphila*. Tian et al. (44) reported that pro-and anti-inflammatory diets are associated with increased abundance of distinct *Bacteroides* species; Lozano et al. (45) reported that a higher energy-adjusted DII is associated with a higher abundance of *Flavonifractor*, *Ruminococcus gnavus* group and *Tyzzerella*, after adjusting for relevant covariates including sex. While this evidence positions diet as potential treatment and prevention targets, results on the association between the inflammatory potential of diet and anxiety are not consistent and specific food- and nutrients combination effects are yet to be confirmed.

As an example of inconsistency, Phillips et al. (46) reported increased anxiety odds in females, but not in males, when comparing the highest to the lowest tertile of energy-adjusted DII. Attlee et al. (47) observed increased anxiety odds in 260 undergraduate females in their categorical analysis and observed positive associations when analysis DII as a continuous variable. Salari-Moghaddam et al. (48) and Salari-Moghaddam et al. (9) reported that a higher DII score and a higher food-based DII (FDII), respectively, are associated with greater odds of anxiety. However, contrasting results were reported in sex-stratified analysis: in the former study, the association between DII and anxiety was seen in neither men nor women after model adjustment; in the latter, association between FDII and anxiety was seen in women but not in men. In a recent cross-sectional study, Varaee et al. (49) reported a positive association between DIS and anxiety after adjusting for confounding factors including sex. Dehghan et al. (7) found no associations between DII categories and anxiety in female adolescents, although the lack of an age-adapted anxiety measure raises concerns of validity and reliability (50). The DII is calculated on a scale, but both studies treated DII as data-dependent categories which might be based on unrealistic assumptions, sacrifice data, mislead analysis and results interpretation. This could hinder studies comparison and should be replaced by, or come along with, continuous methods. The latter would indeed preserve the nature of the data, allow for higher accuracy and sensitivity, while categorical methods could benefit data visualization and interpretation (51). Further considerations include that the DII reflects the diet pro-inflammatory *potential* whose manifestation might depend on subjective physiological mechanisms such as liver function (52), hormonal balance (53, 54), HPA-axis reactivity (55), other than the individual microbiome signature (56). Age-specific effects might also occur due to compensation of homeostatic changes that could remain latent while gradually shaping developmental trajectories toward disease later in time. For example, prolonged diet-driven inflammation might perturb the HPA-axis in an age-dependent manner (57) and increase perceived stress (58). Stress-related neurochemical (59) and gut microbiome (60) changes could then account for an increased anxiety risk. Finally, attention should be paid on how the DII and DIS are computed: whereas nutrients-based DII allows for an in-depth analysis of the food matrix contribution, FDII and DIS account for food patterns and choices (e.g., distinguishing between refined versus whole grains, protein source) thus are more interpretable and reflect the synergic nature of diet at a higher hierarchical level (see Figure 3) Therefore, whereas the DII would best inform the dietary supplements manufacturing, the FDII and DIS might have higher ecological validity and then be more easily translated into public recommendations. More studies employing FDII and DIS should then be carried out while DII components and overall score should be empirically calculated and validated against inflammatory biomarkers.

#### 3.2.2 Healthy eating index and alternative healthy eating index

The Healthy Eating Index (HEI) was developed to score adherence to the *Dietary Guidelines for Americans* (DGA) (39) and multiple editions exist to reflect the evolution of dietary guidance as expressed by the DGA. According to scientific advancements, the last HEI versions focus on diet quality as it arises from a combination of adequacy and moderation of food- and nutrient-based components described elsewhere (61). Associations have been reported between the HEI-2005, HEI-2010, and HEI-2015 with the gut microbiome composition (33, 62, 63). For instance, Liu et al. (64) reported that the HEI-2005 score is positively correlated with *Roseburia* and *Subdoligranulum*, and negatively correlated with *Tyzzerella* in a prevalently male sample. Ma et al. (33) reported that the HEI-2015 score is inversely associated with *Collinsella* and *Tyzzerella*, after adjustment for relevant covariates including sex. Associations have also been reported between the HEI component scores and gut microbiome composition: Liu et al. (64) reported that the HEI-2005 component 2 (whole fruit, no juice) and the HEI-2005 component 7 (milk and soy beverages) are negatively correlated with *Bacteroides* and positively correlated with *Faecalibacterium*, and the HEI-2005 component 12 (solid fats, alcoholic beverages, and added sugars) is negatively correlated with *Escherichia*; Little et al. (65) found that the HEI-2010 component score for fat intake is inversely associated with *Prevotella* and *Escherichia* in adult females. As a modification of the HEI, the Alternative HEI (AHEI) was introduced by Chiuve et al. (37) to rate foods based on disease risk and chronic illnesses. Yu et al. (66) reported that, after adjustment for relevant covariates including sex, a long-term diet quality score that significantly correlated with the AHEI, is positively associated with the abundance of certain gut bacteria. Specifically, this includes the genera *Coprococcus*, *Bifidobacterium*, and *Faecalibacterium*, and the species *Bifidobacterium adolescentis* and *Faecalibacterium prausnitzii*.

Research exploring the relationship between the HEI and anxiety is limited, with slightly more studies investigating the association using the AHEI framework. Of the work available, some authors reported that anxiety – alongside sleep and depressive symptoms – are the only premenstrual symptoms’ subscales that significantly differ between HEI groups in young adolescents. Specifically, the study indicated that higher HEI-2010 scores are associated with decreased anxiety (67). In two other studies, no results were found for HEI scores and anxiety in females (68) nor males (30). When adopting the AHEI, Gibson-Smith et al. (69) found significant results for anxiety severity but not diagnosis, while Saneei et al. (70) reported negative associations between AHEI and anxiety incidence in females but not in males when accounting for potential confounders. In interpreting such results, a few critical points should be accounted for. Firstly, Christensen et al. (68) grouped subjects based on unclear anxiety categories that deviate from conventional cutoff points (71): members from distinct categories have been merged possibly neglecting important information and decreasing sensitivity. Secondly, in the study by Taylor et al. (30) the extended version of the same anxiety scale was used continuously, yet data predominantly clustered at the lower end, raising concerns that may obscure true variability. Further, both studies were characterized by limited sample sizes and lacked preliminary stratification of subjects based on, e.g., HEI grades (72). This might have resulted in undistinguishable population groups (51), potentially hiding existing effects. In summary, promising results have been reported for both the HEI and AHEI although additional research is needed due to the presence of many confounding variables, lack of knowledge about HEI and AHEI differences in anxiety prediction, and absence of studies employing the most recent HEI version, i.e., HEI-2020. Testing the predictive value of the HEI-2020 for anxiety would come with several advantages. First, the HEI is based on clear and periodically revised guidelines and would then ensure an up-to-date framework of reference and DQ proxy. Second, it encompasses nutrient- and food-derived components, thus equally integrates multiple hierarchical levels for a more comprehensive assessment. Third, single component scores can be analyzed both individually and collectively to reveal specific effects and patterns of dietary quality (72). The HEI would then easily allow multiple-levels dietary analysis in a systematic and reproducible way. A funnel-shaped analysis should be preferentially adopted in epidemiological research: no simple models could in fact answer complex and multifactorial questions. Rather, simultaneously looking at single dietary variables, and within- and between-levels interactions would help nail down the differential weight exerted by individual dietary components and synergic interplays.

#### 3.2.3 The dietary diversity score

The dietary diversity score (DDS) is a food-derived index, developed by the Food and Agriculture Organization of the United Nations, that reflects diet variety at the household or individual level (73). Diet variety refers to the number of different foods or food groups consumed over a given reference period. Interestingly, controlling for confounding effects including sex, a more vary diet as assessed by the DDS and Dietary Variety Score has been associated with increased alpha diversity in gut microbiome and decreased *Roseburia* abundance (74, 75). The DDS has been found to be negatively associated with anxiety in adult female samples cross-sectionally (76) and longitudinally (77), although food-specific correlations were inconsistent both within and between studies. The latter could be justified by the adoption of different food groups. For example, Jiang et al. (77) distinguish meat from fish/sea food whereas Poorrezaeian et al. (76) do not. Ambiguities in categorization methods also arise from unjustified different cut-off choices: Poorrezaeian et al. (76) adopt a threshold of 3 to distinguish between low and high dietary diversity, Jiang et al. (77) set this boundary at 6. When clear classification systems do not exist, arbitrary cut-off points should be discouraged while research is carried out to validate and compare scoring systems so to improve systematicity and reproducibility of existing diet quality indexes (78).

#### 3.2.4 Other diet quality indices

Several other composite measures have been used to assess the influence of diet quality on anxiety and the findings will be briefly summarized here. Mobarakeh and Eftekhari (79) found higher scores of Diet Quality Index – international (DQI-I) to predict lower anxiety in a sample of Iranian females. Although the DQI-I is a highly comprehensive score built on dietary variety, adequacy, moderation, and overall balance recommendations, it’s intended, and might present higher sensitivity for, cross-national comparison (80). Another study found the same results using the Food Quality Score (FQS) that was computed by ranking 14 food items based on their “favorable vs. unfavorable” effect on weight health (81). However, what’s healthy for weight management is not necessarily healthy for mental health. For example, whereas coffee was included in the “favorable” category, research seems to suggest that coffee worsens anxiety in a dose-dependent manner (5). The Recommended Food Score (RFS) has also been developed to measure food derived DQ but has not been associated with anxiety severity (82). A few nutrient-derived DQIs also exist and have been negatively associated with anxiety, namely the Dietary Phytochemical index (DPI) (83, 84), the Dietary Antioxidant index (7), and the Dietary Antioxidant Quality Score (DAQS) (85). Sangsefidi et al. (85) observed a negative association between DAQS and anxiety in females (but not in males), a relationship that became non-significant after adjusting for body mass index. The Dietary Total Antioxidant Capacity has also been negatively associated with anxiety in postmenopausal Iranian women (86, 87). Conversely, dietary acid–base load indexes have been positively associated with anxiety in Iranian women (88). It is worth noting that both phytochemical and antioxidant intake have been linked to gut microbiome. Various classes of phytochemicals were reported to decrease the Firmicutes/Bacteroidetes ratio and to increase the gut microbiota diversity (89). Caffeine, which may be one of the most prominent alkaloids consumed, was associated with a higher gut microbiome diversity and an increased abundance of *Faecalibacterium* and *Roseburia* species (89). In an *in vitro* study, an extract of green tea, which contains phenolic compounds responsible for its antioxidant capacity, was shown to inhibit the growth of *Escherichia/Shigella* and increase the growth of *Faecalibacterium* and *Roseburia* (90). Finally, research suggests a two-way relationship between antioxidant intake and the gut microbiome: antioxidant foods regulate gut microbiome homeostasis, and antioxidant bioavailability is influenced by metabolites produced by gut microbes (91).

### 3.3 Dietary patterns

While DQIs score diets against recommendations and/or evidence-based knowledge, dietary patterns consider the combination of food and/or nutrients. It is worth highlighting that adherence to distinct dietary patterns could potentially present similar DQIs. For example, the Mediterranean and Nordic diets both emphasize the consumption of local and seasonal food and are based on similar principles that align to the GDA, thus likely to be associated with high HEI. In computing dietary patterns, two approaches exist and will be separately discussed: *a priori* methods based on index-based patterns, and data driven methods based on exploratory analysis.

#### 3.3.1 Index-based patterns

*A priori* methods rely on pre-determined dietary standards and/or country-specific eating clusters. A study investigating the Nordic diet including high consumption of wholegrains, fruits, vegetables, fatty fish, and legumes, found no effect of overall diet on anxiety in female university students, but an inverse relationship with cabbage consumption (92). Similarly to the Nordic diet, the Mediterranean diet (MD) is traditionally characterized by high consumption of vegetables, fruits, nuts, legumes, wholegrains, and moderate intake of processed meat and short-preservable cheese (93). The MD has been associated with a higher gut microbiome diversity and increased abundance of *Faecalibacterium prausnitzii* (94, 95). Ruiz-Saavedra et al. (96) identified, in a sample of prevalently Spanish adult females, the DII, HEI, the Mediterranean adapted Diet Quality Index International, and the Modified Mediterranean Diet Score as predictors of *Faecalibacterium prausnitzii*, with higher levels in individuals with healthier diets. The MD has been associated with health benefits in many conditions (97) including mental health. Accordingly, anxiety severity, but not diagnosis, was found to be inversely associated with MD adherence, as measured by the MedDiet score (69). In this study, no additional analysis was performed to investigate associations between Mediterranean food staples and psychological outcomes. Adapting the same Mediterranean score to a more culturally heterogenous population, Boaz et al. (14), found similar results in both males and females while pinpointing sex-specific and food items-specific associations. In both sexes, the authors found positive associations with butter/margarine/cream, red/processed meat, savory baked goods, salty snacks, and sweetened beverages; negative associations with legume-based dips. In females only, anxiety was positively associated with wholegrains, legumes, and alcoholic beverages; negatively associated with olive oil as main culinary fat, vegetables, and unsweetened dairy. In males only, anxiety was negatively associated with fish and nuts intake. Similarly, Sadeghi et al. (98) reported that habitual consumers of the Mediterranean diet, as evaluated by the MDScale index, exhibit a lower risk of anxiety. Further, they reported that higher intake of vegetables and fruits predicts a lower anxiety risk, grains consumption a higher risk. Despite the promising findings, it becomes clear that there is no unified vision of food components that should be used to assess adherence to the Mediterranean diet and that several indices exist (99). Critically, the grains category in the MDScale do not distinguish between refined and whole grains which seem to have opposite effects on anxiety in females (100) and some Mediterranean dietary indices, including the MDScale, only refer to adequacy standards while neglecting moderation guidelines. Specificity and sensitivity differences might also exist between food frequency questionnaires employed by authors evaluating the MD due to the number of items included. For example, Gibson-Smith et al. (69) and Sadeghi et al. (98) administered a 238-item and 106-item FFQ, respectively, Boaz et al. (14) used a 17-item questionnaire which may have lower sensitivity.

While both Nordic and Mediterranean diets promote a high intake of plant-based food, vegetarian and vegan diets focus on this exclusively. Notably, individuals following plant-based diets showed higher HEI-2010 scores compared to omnivores (101). Furthermore, Deng et al. (102) found that vegetarian adult women exhibit greater gut microbiota richness than their omnivore counterparts. They identified *Tyzzerella 3* as an enriched species in vegetarians, suggesting it as a potential discriminator between the two dietary groups. However, they also reported that changes in the gut environment diminish over time with prolonged adherence to a vegetarian diet. Contradictory results exist for anxiety levels in vegetarian and vegan populations. For example, Michalak et al. (103) found that vegetarians displayed higher anxiety rates than omnivores. Beezhold et al. (104) observed lower anxiety in vegetarian men, but not women, compared to omnivores, with vegan men also showing reduced anxiety. However, when including data from a pilot study, both male and female vegans reported less anxiety than omnivores. It is interesting to note that adherence to a vegan diet was positively associated with lower stress in females only, hinting at distinct sex-related mechanisms engaging the HPA axis on a different level. While these findings are encouraging, they require cautious interpretation to avoid attributing significance to potentially spurious correlations. Factors such as vegans “spending more time outdoors, exercising more, having a lower alcohol intake, consuming fewer sweet servings per day, and being older” are indeed closely associated with decreased anxiety and were not controlled for in the statistical tests.

#### 3.3.2 Exploratory patterns

Exploratory dietary patterns (DPs) rely on *a posteriori* approach that derives dietary food and/or nutrients patterns from the collected data. Investigations on the relationship between DPs and gut microbiome have yielded several findings. Ericson et al. (105) found that adherence to a “health-conscious pattern” is associated with a higher abundance of *Roseburia*. Malinowska et al. (106) observed that a healthy dietary pattern is associated with a higher abundance of *Faecalibacterium* and a lower abundance of *Escherichia-Shigella* compared to a Western dietary pattern. Turpin et al. (107) reported that a dietary cluster resembling the Mediterranean diet is associated with an increased abundance of *Faecalibacterium*. As regards anxiety, Rossa-Roccor et al. (108) did not find any effect of either the plant- or the animal-based dietary pattern after adjusting for relevant covariates. However, the authors reported a positive association between the junk food dietary pattern and anxiety. Interestingly, the magnitude of the effect was comparable to other covariates known to strongly correlate with mental health outcomes, such as social support and stressful life events. Bakhtiyari et al. (109) also showed that higher processed food intake predicts higher trait and state anxiety in young adults, contrarily to what Vilela et al. (110) found in a prospective cohort study of pregnant women: although they found higher anxiety in women with higher adherence to a processed DP, the association was not significant when performing multivariate regression. The same authors found discordant results for “traditional Brazilian” and “healthy” patterns as did Yazdi et al. (111) and Xu et al. (112): “Western” dietary patterns were shown to predict higher anxiety as opposed to “healthy” and “grains-vegetables” clusters, whereas no significant results were found for “traditional” (111, 112) and “high-salt” (112) DPs. In the study conducted by Weng et al. (113), the traditional dietary pattern – a typically healthy and recommended diet – was associated with decreased odds of anxiety, albeit not significantly. The same authors also reported the snack and animal food patterns to be associated with higher odds of anxiety. Finally, Hosseinzadeh et al. (114) did not find any effect of “lacto-vegetarian,” “Fast food” nor “Western” DPs on anxiety in Iranian females, but positive effects of a “traditional” DP; absence of effects was found in males. Probably due to cultural differences, “Traditional” patterns across studies do appear arbitrary and only partially overlapping. For example, Vilela et al. (110) includes rice, beans, meats and eggs, and vegetable spices. Yazdi et al. (111) includes high fat dairies, red meat, poultry, bread, rice, potatoes, fried food, hydrogenated vegetables oils. Xu et al. (112) encompasses whole grains, vegetables, fruits, mushrooms, poultry and organs, fish, egg, soya products, vegetable oil and tea. The same applies to “healthy” DPs which include pasta, cakes, cookies-crackers, and candies, which are normally categorized as processed food, alongside tubers, vegetables, and other health-promoting foods in Vilela et al. (110) study. These food items differ from those included by Yazdi et al. (111), namely dairy, fish, fruits, fresh fruit juice, veggies, beans, soy protein, nuts, garlic, and non-hydrogenated vegetables oils. Such inconsistencies could explain different results across studies and question the validity and comparability of exploratory DPs. The latter may be preferable when well-defined and standardized definitions are lacking, when dealing with large sample sizes, or for conducting exploratory analyses to periodically validate and update existing dietary knowledge and guidelines. On the other hand, definitions are challenging to agree on mostly due to existing overlaps. For example, processed plant-based food falls into both the junk and plant categories. In the study conducted by Rossa-Roccor et al. (108), the processed and ultra-processed plant-based food did strongly load to the plant component potentially diluting the health-promoting effects of real plant food. When possible, good practice should then adopt dietary measures that accommodate the interplay between food source and quality. Consistent with this, Mousavi et al. (115) found opposite effects of plant-based food on anxiety when distinguishing between healthy and unhealthy choices. Such findings raise concerns about existing definitions and highlight the urge for periodical reassessments to provide an up-to-date framework for researchers to align with.

While most of the research focused on food-derived dietary patterns, Salehi-Abargouei et al. (116) adopted a nutrients-based approach. They found that men, but not women, who closely followed an omnivore nutrient pattern rich in individual amino acids, cobalamin, zinc, phosphorus, saturated fatty acids, cholesterol, and pantothenic acid, had lower anxiety scores. However, this association disappeared under multivariate logistic analysis. Worth noting, women adhering to this dietary pattern showed reduced psychological distress, association found also when adjusting for multiple confounders in logistic models. Contrarily to food patterns, nutrient patterns cannot capture differences in availability and absorption. For example, differences in the molecular structure of plant- and animal-derived amino acids affect their digestibility and bioavailability rates. It follows that amino acid levels in two different individuals with similar dietary intake could yet differ based on *food* choice leading to confounding results and erroneous conclusions. Food and nutrients could also be combined to build overall DPs. Interestingly, Cotillard et al. (13) investigated dietary-gut microbiome associations by comparing multiple dietary operationalization strategies and found overall DPs to present with more significant associations than single dietary components. Food-derived DPs or combined food- and nutrients-derived DPs could then be a more reliable measure able to integrate both nutrients’ source and synergic effects and further studies should be conducted to investigate their association with anxiety.

### 3.4 Single foods and food groups

Analyzing diet quality through DPs presents certain drawbacks: firstly, it neglects the unique contribution of individual foods to the observed effect and the fact that different combinations of foods can result in similar DPs. Second, it can dilute the impact driven by a specific subset of foods. Food groups and single food could be conceptualized as the foundational units of food-derived DPs. Most research has been conducted on fruit and vegetable intake, identifying them as key factors in enhancing gut microbiome diversity and composition due to their high content of polyphenols and fibers (117). An interventional study in healthy adults matched for sex reported higher abundance of *Faecalibacterium* in individuals consuming a fruit and vegetable supplement compared to those consuming a placebo (118). Galena et al. (119) found that after six weeks of consuming fermented vegetables, women exhibited increased levels of *Faecalibacterium prausnitzii* and *Roseburia faecis*. This change was observed neither in women who consumed pickled vegetables nor in the control group. Regarding the effects of fruit and vegetable intake on anxiety, inconsistent results have been reported. Saghafian et al. (120) reported fruits only to predict lower anxiety in females, whereas vegetables only in males. Differently, Boaz et al. (14) found associations between vegetables intake only and decreased anxiety in females, and no significant results in males. Gibson-Smith et al. (121) and Sadeghi et al. (98), in analyses adjusted for sex, found that consuming solely vegetables and both fruits and vegetables, respectively, predict decreased anxiety. No effects were found in either male, female, or mixed samples when clustering fruits and vegetables together (120, 122, 123) or when distinguishing between raw and processed intake (124). An interventional study also observed that tomato-juice alleviates anxiety, although no control group was included (125). A recently published systematic review reported that anxiety-related symptomatology is improved by fruit and vegetable consumption, but these effects are small and imprecise; most importantly, the evidence available to draw conclusions is extremely limited (126). Some authors looked at grains intake and reported a negative association between wholegrains consumption and anxiety (121). When distinguishing between sex, Sadeghi et al. (100) observed positive versus negative associations of refined- and whole-grains, respectively, in females, and no significant results in males. Differently, Boaz et al. (14) found positive associations between wholegrains and anxiety in females only, while Abbaszadeh et al. (92) no significant results. Interestingly, a higher whole grain intake has been associated with a higher abundance of *Faecalibacterium prausnitzii* and *Roseburia*, after adjusting for relevant covariates including sex (127). In a prospective cohort study in Thai pregnant women, Phoonlapdacha et al. (128) observed that glutinous rice consumption had a mixed impact on gut bacteria. Specifically, it was positively associated with the Bacteroidetes phylum and negatively associated with the Firmicutes phylum. At the genera level, positive associations were found for *Bacteroides*, negative associations for *Prevotella*, even though both genera are part of the Bacteroidetes phylum. Similarly to refined grains, savory snacks and foods high in added sugars are prevalent in Western dietary patterns and have been observed to predict increased anxiety (122, 123, 129). Some authors also looked at red meat intake and consistently observed positive associations with anxiety in females (14, 129–131) whereas conflicting results have been found in males (14, 129). Interestingly, a greater consumption of processed meat has been associated with lower Shannon and Simpson indices, and reduced *Roseburia* abundance in adolescents, after adjusting for relevant covariates including sex (132). Consistently, long-term intake of processed meat was shown to negatively affect *Roseburia* and *Roseburia faecis* abundance, with adjustments for sex and other covariates (66). Regarding health-promoting foods, Boaz et al. (14) observed a positive association between legume intake and anxiety in women but not in men. Differently, Anjom-Shoae et al. (133) found that a combined intake of legumes and nuts was linked to lower odds of anxiety in men, but this association was not seen in women. Reeder et al. (134) conducted an interventional study comparing skin roasted peanut to a peanut-free group and found no significant differences. In contrast, Parilli-Moser et al. (135) observed reduced anxiety in consumers of skin roasted peanuts, unlike those consuming peanut butter, compared to a group consuming peanut oil devoid of phenolic compounds and fibers. Nut consumption was also shown to affect gut microbiome composition. An interventional study showed that walnut consumption increases *Faecalibacterium* and *Roseburia* abundance in both men and women (136). A parallel study from the same team also revealed that almond processing affects *Roseburia* levels differently: roasted chopped almonds significantly increased its abundance, both whole roasted and natural almonds showed a positive increasing trend, almon butter showed no effects (137).

### 3.5 Single nutrients and nutrients group

While DPs can be viewed as combinations of single food and food groups, it is also accurate to consider them as emerging from the food matrix, i.e., the food microstructure that accommodates nutrient interactions, functions, and behavior beyond isolated nutrients. As previously mentioned, the literature of nutrient-derived DPs is lacking, and most studies have focused on nutrient groups and single nutrients. Research focusing on macronutrients intake and anxiety has yielded inconclusive results: studies have found no significant effects of following a low carbohydrate diet (138, 139), using a carbohydrate quality index (140), or considering glycemic index and load (141). No significant effects on anxiety were observed with a protein quality index in Hajihashemi et al. (140); however, the method of calculation is debatable as computed based on foods overall rather than their protein content. Studies looking at single amino-acids reported inconsistent results of lysine, lysine/arginine, and alpha-lactalbumin interventions (142–144). Differently, anxiety decrease was observed in associations with fat quality, computed as ratio between unsaturated and saturated/trans fatty acids. The association remained significant after adjustment for multiple factors only when a higher anxiety cut-off point was considered (140). Unsaturated fats, including mono- (MUFAs) and poly-unsaturated fatty acids (PUFAs), are predominantly found in plants and fish and have several health benefits (145), whereas the role of saturated fatty acids (SFAs), found in animal foods and tropical oils, is still controversial (146). Trans fats do not occur naturally but are created during industrial processes to make food more desirable and palatable with deleterious effects on health (147). Research investigating trans-fatty acids effects on anxiety is missing, but one study showed a positive association with negative affect (148), a factor previously reported as predictor of anxiety disorders (149). Ford et al. (148) found no associations with either n-3, n-6, nor n-6-n-3 PUFAs ratio, similarly to Watanabe et al. (150) who found no effect of n-3 PUFAs on anxiety following a 13-week randomized-controlled intervention in females. A reduction in anxiety was noted at a 52-week follow-up in the latter study, though the authors caution that this finding may result from multiple testing. Daley et al. (151) did also report no effect of individual nor total n-3 PUFAs in a large cross-sectional study of young females. However, they observed decreased anxiety associated with linoleic acid only and total n-6 PUFAs intake, while increased anxiety predicted by n-9 MUFAs intake. Opposite findings have been shown by other authors reporting inverse associations between anxiety and dietary intake of n-3 eicosatetraenoic and docosahexaenoic fatty acids but not n-6 linoleic and arachidonic fatty acids in young female athletes (152). Decreased anxiety was also shown following a 12-week n-3 PUFAs supplementation by Kiecolt-Glaser et al. (153) who further observed associations between higher anxiety and increasing blood n:6-n-3 ratio. Worth noting, such effects were found despite low anxiety scores at baseline, which is a risky condition for floor effects. Many foods high in fatty acids are also enriched in bioactive compounds. For example, peanuts contain high levels of MUFAs, fibers, as well as polyphenols that may contribute to decreasing anxiety levels (135). A high-flavonoid food-based intervention was also shown to improve state, but not trait, anxiety (154), differently from a study where polyphenols supplementation through concord grape juice yielded no results (155). Some authors focused on fibers and found significant improvements in anxiety predicted by higher dietary fibers intake in females but not in males after adjusting for relevant factors (156). Improved anxiety symptoms were also seen after a 4-week 7.5 g/d of galacto-oligosaccharide intervention (29), but not polydextrose supplementation (157) in female samples. Variations in the outcomes may stem from the type of oligosaccharides used: galacto-oligosaccharides are naturally found in foods, while polydextrose is synthetically produced. Additionally, Johnstone et al. (29) utilized a larger sample size and a more targeted age range. Interestingly, fiber intake was shown to also affect the composition of the gut microbiome. Adamberg et al. (158) reported that a higher fiber intake is associated with increased abundance of, e.g., *Roseburia hominis* and *Bacteroides xylanisolvens* in healthy Estonian adult men and women, while fiber-deficient diets are associated with, e.g., *Bacteroides coprocola* and *Collinsella aerofaciens*. Gomez-Arango et al. (159) studied the impact of fiber consumption in overweight and obese pregnant women, finding that low fiber intake was associated with higher levels of *Collinsella* and *Prevotella*. Even after adjusting for total energy intake, low fiber intake remained associated with *Collinsella*, whereas high fiber intake was associated with increased *Faecalibacterium* and *Roseburia*.

Finally, little research has been performed on effects of micronutrients supplementation on anxiety and no significant results were reported for a multivitamin complex nor vitamin E, C, and zinc alone (160–162), inconsistent effects for magnesium (160, 163), and ameliorating effects for b-carotene and iron-fortified micronutrient supplementation (161, 164).

## 4 Diet, gut microbiome, and anxiety: integrating available evidence for translational opportunities

### 4.1 Overview of underlying interplays

**Figure 4:**
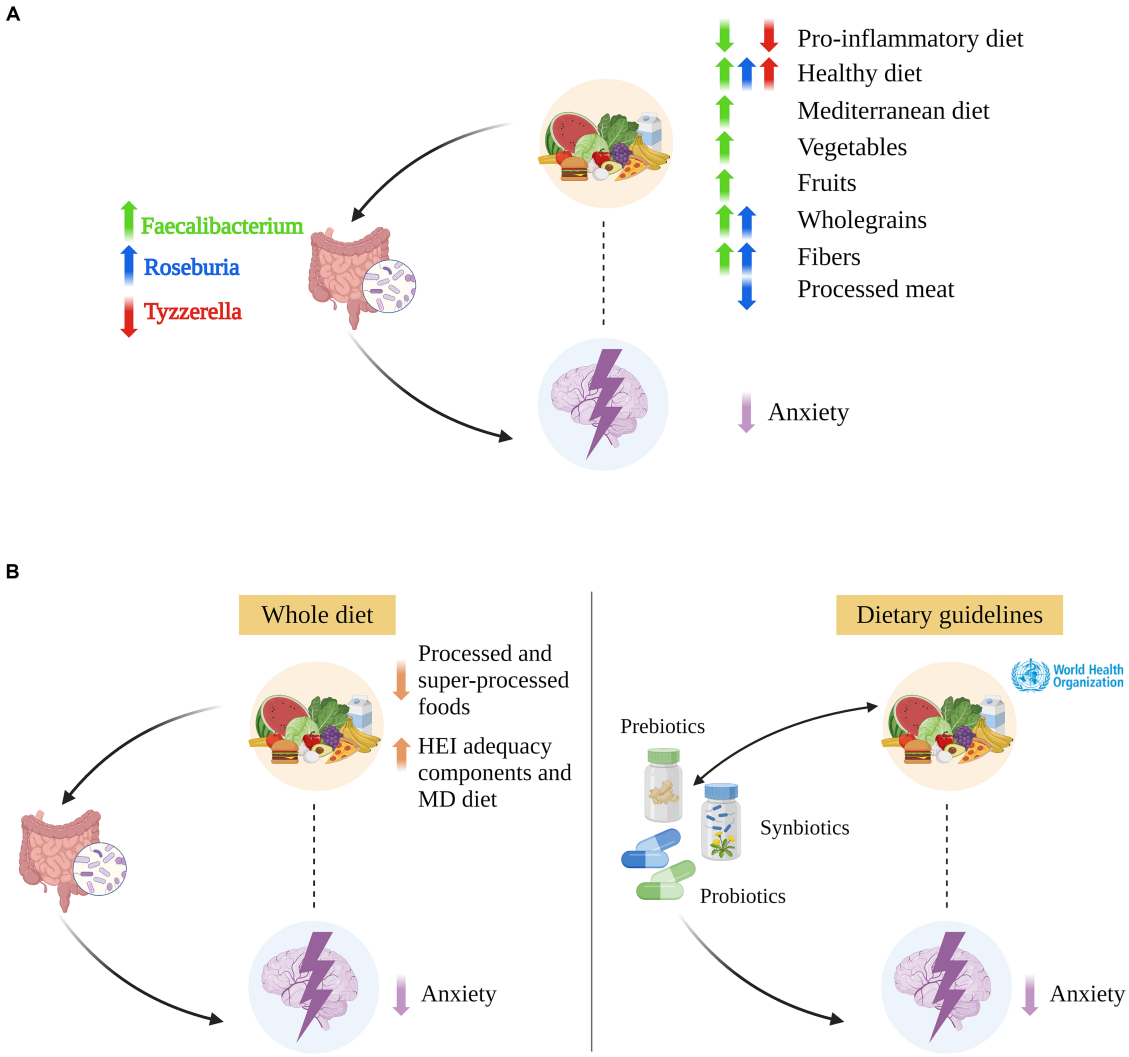
Integration of diet, gut microbiome, anxiety, and translational opportunities. **(A)** The integration of available evidence on relationships among diet, gut microbiome, and anxiety suggests that some bacterial genera such as *Faecalibacterium*, *Roseburia*, and *Tyzzerella* likely play a mediatory role in the relationship between diet and anxiety. Only more relevant relationships cited in the main text are indicated. ↑ (↓) indicates an increased (decreased) genus abundance; an increased (decreased) adherence to a pro-inflammatory, healthy, and Mediterranean diet; and an increased (decreased) consumption of vegetables, fruit, whole/non-refined grain, fibers, and processed meat. **(B)** Left: The interconnection among diet, gut microbiome, and anxiety, and the mediatory role we propose for the gut microbiome, suggesting that whole diet interventions may alleviate anxiety symptoms by favoring or inhibiting the growth of definite bacterial species. Right: Probiotics, prebiotics, and synbiotics interventions could reduce anxiety by modifying the gut microbiome composition, for example for *Faecalibacterium* and *Roseburia* [in green and blu color, respectively, in panels **(A,B)**]. In combination with high adherence to a healthy diet, they could foster reciprocal benefits, enhancing overall efficacy. Created with and adapted from BioRender.com.

As previously mentioned, research looking at diet, gut microbiome and anxiety simultaneously is scarce. A few studies investigated microbiome-anxiety specific associations yet reported contradictory results. For example, Chen et al. (165) and Jiang et al. (166) conducted two case–control studies on individuals affected by generalized anxiety disorder and found that, while gut microbiome richness significantly differed from that of healthy controls, there were no notable differences in the Shannon or Simpson diversity indices. No sex-stratified analysis or adjustment were performed, yet between-samples diversity analysis revealed that gut microbiome composition was significantly associated with sex in Chen et al. (165) but not in Jiang et al. (166). The discrepancies in findings may be attributed to the specific subgroups analyzed: Jiang et al. (166) focused exclusively on anxious patients, while Chen et al. (165) considered the entire sample. Both studies also analyzed compositional differences and reported consistent results. Anxious patients showed higher abundance of *Bacteroides* (Bacteroidetes phylum), *Escherichia/Shigella* (Proteobacteria phylum), *Tyzzerella* (Firmicutes phylum), and lower abundance of *Subdoligranulum, Faecalibacterium*, and *Roseburia* genera (Firmicutes phylum) as summarized in Chen et al. (167) and Simpson et al. (168) (see Figure 4A). In a study examining anxiety symptomatology without specifying clinical diagnoses, no difference in alpha diversity metrics was observed between women with and without anxiety symptoms, as assessed by the Beck Anxiety Inventory (BAI). However, this finding was sex-specific, with reported differences in males (169). The authors also showed lower relative abundance of *Prevotella* (Bacteroidetes phylum) in anxious female participants. Likewise, Ganci et al. (16) identified sex-specific relationships in females, noting both positive and negative associations between anxiety symptoms and specific Firmicutes species, including negative associations with *Clostridium innocuum, Enterococcus durans, Leuconostoc lactis, Ruminococcus gnavus* and positive associations with *Lactobacillus paracasei*, and *Streptococcus dysgalactiae*. In a study investigating positive and negative affect, Lee et al. (35) found that *Collinsella* (Actinobacteria phylum) was inversely associated with positive affect, whereas a new genus from the Lachnospiraceae family (PAC001043) showed a positive association with positive affect and a negative association with negative affect. Conversely, Kleiman et al. (170) found no significant associations between anxiety and either microbiome composition or diversity in healthy females. It is important to highlight that Kleiman et al. (170) included participants displaying “normal” or “minimal levels of anxiety and psychiatric measures” thereby excluding individuals with subclinical levels of anxiety who were included in the study by Ganci et al. (16) and Kim et al. (169). Conflicting results may be due to a non-linear relationship between microbiome and anxiety across different levels of anxiety severity. Alternatively, when categorizing anxiety risk in (i) no anxiety, (ii) sub-clinically anxious, and (iii) clinically anxious, the strength of associations may vary among categories.

Dietary effects on gut microbiome have been extensively investigated, and several studies proving evidence for diet influence on anxiety-associated gut microbes have already been cited in Section 3 (see Figure 4A). In addition, Ma et al. (33) observed that, after controlling for covariates including sex, the HEI-2015 is negatively associated with *Bacteroides, Escherichia/Shigella, Tyzzerella*, *Collinsella, Odoribacter* (Bacteroidetes phylum); positively associated with *Faecalibacterium.* The HEI-2015 also showed mixed associations, either negative or positive, with 24 other genera, predominantly within the Lachnospiraceae and Ruminococcaceae families of the Firmicutes phylum. In a predominantly male sample, Liu et al. (64) found that HEI-2005 components are associated with specific gut microbes’ changes. For instance, the authors reported that the HEI-2005 component 2 (whole fruit, no juice) and the HEI-2005 component 7 (milk and soy beverages) are negatively associated with *Bacteroides* and positively associated with *Faecalibacterium.* They also noted the HEI-2005 component 12 (representing added sugars, alcohol, and saturated fats) to be associated with increased *Escherichia* and decreased *Subdoligranulum*. In adult females, Little et al. (65) observed an inverse relationship between the HEI-2010 fat intake score and *Escherichia* abundance and identified that both saturated and unsaturated fats are positively associated with *Odoribacter*. Contrasting with Little et al. (65), Berding et al. (12) outlined distinct associations between dietary fats and gut microbiome in their review. They linked MUFA/PUFAs with increased beneficial bacteria such as *Roseburia, Bifidobacterium*, and *Lachnospira*; while SFAs with decreased bacteria diversity and increased abundance of proinflammatory bacteria including *Alistipes* and *Ruminococcus gnavus*. Similarly, Watson et al. (171) reported that an omega-3 PUFA intervention delivered in a drink form (but not as capsules) reversibly increased abundance of *Roseburia*, with no sex-related differences observed. Garcia-Montero et al. (36) reported n-3 PUFAs to be associated with increased *Prevotella* and *Ruminococcus* and to balance Bacteroidetes/Firmicutes ratio. They also reported n-6/n-3 PUFAs ratio to be positively associated with abundance of Enterobacteriaceae and Clostridia classes. Wan et al. (172) compared low (20%), medium (30%) and high (40%) fat diets showing increased Shannon index and *Faecalibacterium* abundance in the lower fat diet. A high-fat diet resulted in increased *Bacteroides* abundance and decreased concentration of fecal short-chain-fatty-acids (i.e., gut bacteria by-products) alongside elevated levels of plasma C-reactive protein. It is worth noting that the main fat source included in the intervention was soybean oil, primarily consisting of PUFAs, predominantly omega-6 fatty acids. Finally, research extends beyond SFAs to examine the impact of other Western diet components such as TFAs, added sugars, refined oils, processed meat. Garcia-Montero et al. (36) summarized findings indicating that these elements all affect anxiety-associated gut bacteria. For example, high consumption of added/free sugars is linked to increased Firmicutes/Bacteroidetes ratio and decreased butyrate-producing bacteria. Likewise, TFAs promote gut dysbiosis by negatively affecting butyrate producers. Butyrate is a key short-chain fatty acid mainly produced by certain Firmicutes genera such as *Faecalibacterium*, *Subdoligranulum*, and *Roseburia*, previously mentioned to be decreased in anxious groups. While the evidence is not yet definitive, preliminary results are promising, suggesting the need for further research into how diet influences anxiety through alterations of the gut microbiome.

### 4.2 Translational opportunities: whole diet, probiotic, and prebiotic interventions

The close interconnection among diet, gut microbiome, and anxiety, as well as the mediatory role we propose for the gut microbiome, lays the ground for the development of interventions that could reduce anxiety by modifying the composition of the gut microbiome (Figure 4B). Whole diet interventions that minimize the intake of processed and super-processed foods and maximize the intake of HEI adequacy components, such as whole grains, vegetables, and fibers, represent the first line of intervention. These may alleviate anxiety symptoms by favoring or inhibiting the growth of definite bacterial species within the Firmicutes and Bacteroidetes phyla, and they may disrupt the vicious cycle of emotional eating. Noteworthy, whole diet interventions could benefit both clinical and community settings, possibly representing a preventive measure to halt the rising incidence of anxiety. Unsurprisingly, participants compliance is a common limitation of whole diet intervention studies, especially when these are carried out over a long period of time. Moreover, when looking at the effects of Western-like components, placebo-controlled trials are hardly implementable due to ethical considerations.

When anxiety is severe and/or the anxiolytic effects exerted by a healthy and balanced diet are mild, probiotic and prebiotic supplements may be used. Probiotics are live bacteria with health-promoting activity and capable of releasing neuroactive substances. For instance, *Lactobacillus brevis* and *Bifidobacterium dentium* were shown to produce GABA *in vitro* (173), and *Lactobacillus rhamnosus* was shown to modulate the expression of GABA receptors *in vivo* (174). *Lactobacillus rhamnosus* was also shown to reduce the levels of stress-induced corticosterone, and anxiety- and depression-related behavior in healthy mice (174). Interestingly, Janik et al. (175) reported that treatment with *Lactobacillus rhamnosus* alters the abundance of neurometabolites such as glutamine + glutamate, total N-acetyl aspartate + N-acetyl aspartyl glutamic acid, and GABA. These changes are comparable with the observed delays in achieving clinical therapeutic effects seen with antidepressants (175) and with anxiolitcs in children and adolescents (176). Prebiotics are non-digestible food ingredients that are selectively utilized by intrinsic beneficial bacteria conferring a health benefit. For instance, milk oligosaccharides were shown to prevent stress-induced gut microbiome changes and anxiety-like behavior in mice (177). Combined GOS and fructo-oligosaccharides (FOS) were shown to exert anxiolytic and antidepressant effects, and to reduce stress and inflammatory responses (178). Interestingly, in the latter study, changes in SCFAs concentration were shown to be correlated with the reported behavioral effects, suggesting a potential mechanism of action.

Although preclinical animal studies yielded promising results on the anxiolytic effects of probiotic and prebiotic supplements, human studies reported inconsistent results. Cohen Kadosh et al. (179) conducted a systematic review and meta-analysis of probiotic and prebiotic interventions on anxiety in youth. Their findings revealed that out of six probiotic studies, five did not find any significant effect, while one reported an improvement of worrying symptoms only in subjects administered with a high daily dose. Out of five prebiotic studies, two found no significant effect, while three reported a decrease in the level of anxiety. More recently, Zhao et al. (180) conducted a systematic review of randomized controlled trials assessing the anxiolytic effect of treatments involving probiotics, prebiotics, or synbiotics – a mixture of probiotics and prebiotics. This work revealed that both probiotics and synbiotics can significantly reduce anxiety scores, while prebiotics do not exert any significant effect compared to placebos. Inconsistencies among trials results may be explained, at least in part, by heterogeneity among studies as regards, e.g., probiotic strains and prebiotic substances, supplements dosage, and indices/scales adopted to measure the level of anxiety. Consistently with that, Zhao et al. (180) found anxiety-scale specific effects, with positive interventional effect for STAI-S and BAI as compared to the DASS-A, the Hospitalized Anxiety Depression Scale, and others. They also reported that high-dose probiotics and multiple-strain supplements have a stronger effect on reducing anxiety scores compared to low-dose probiotics and sole/no-strain supplements, respectively. However, Zhao et al. (180) could not determine the clinical efficacy of probiotic/prebiotic/synbiotic treatments in alleviating anxiety, due to the small combined effect size and the scarcity of studies addressing prebiotics and synbiotics.

The current study highlights the interconnections among diet, gut microbiome, and anxiety, suggesting that probiotic and prebiotic supplementation should always be accompanied by a healthy and balanced diet. Indeed, in terms of translational opportunities, the three-way relationship among diet – gut microbiome – anxiety becomes a three-way relationship among whole-diet interventions (dietary guidelines/programs) – probiotic and prebiotic supplements – anxiety (Figure 4B). Diet and pro-/prebiotic supplements both contribute to decreasing anxiety levels and, at the same time, they influence one another. On one hand, a healthy and balanced diet may enhance the effectiveness of supplements by providing a favorable environment for the growth of probiotic species. Conversely, unhealthy dietary choices may undermine the efficacy of these supplements by acting on the gut microbiome composition and on the same gut-microbiome mediated mechanisms targeted by the supplements (e.g., immune system-mediated inflammation, alteration of the intestinal mucosal barrier). On the other hand, pro-/prebiotic supplements may help disrupt the emotional eating cycle by modulating appetite and influencing foods choice. For instance, Johnstone et al. (181) reported that, in young females, prebiotic GOS supplementation led to changes in nutrient intake such as a decrease in energy from carbohydrates, predicted by increasing *Bifidobacterium* abundance, and energy from sugars. In the same study, anxiolytic effects of GOS supplementation were also reported for high – but not low – anxious subjects (29). In addition, it is worth noting that the baseline microbiome may by itself be a modifier of the effects of diet/supplements on health (32), and hence not all individuals may respond to diet and/or supplement intervention in the same way. For instance, the impact of fibers on colitis susceptibility is highly variable among individuals and may depend on one’s individual microbiome composition (182). Indeed, this study reported that mice transplanted with a fiber-sensitive microbiota, but not mice transplanted with a fiber-resistant microbiota, exhibit exacerbating intestinal inflammation following supplementation with the soluble prebiotic fiber inulin. These results, while not directly informing anxiety outcomes, illuminate the concept that foods may not uniformly benefit everyone and that individual differences in gut microbiome should be considered.

In conclusion, probiotic and prebiotic supplements represent promising opportunities to translate the emerging evidence on the relationship among diet, gut microbiome, and anxiety into clinical practice. As the field advances, further investigations are needed to identify *which* strains are beneficial, their optimal dosage and timing of administration. Moreover, adopting an integrative perspective that encompasses dietary habits, and tailoring interventions to reflect individual differences is strongly advised for optimal outcomes.

## 5 Discussion

A summary of the reported dietary effects on anxiety can be found in Figure 3 and Table 1. The Western diet has shown the most consistent and prominent effects on anxiety so far in both males and females. Increase of anxiety could arise from the combination of foods or be driven by food groups such as red meat, snacks, and sweets. However, no studies have compared their unique contribution to anxiety against their collective effects. Similarly, nutrients found in high concentrations in Western foods (e.g., added sugars and TFAs) play a major role. The connection between observed effects of Western dietary components and their causes remains unclear: the effects could be directly attributed to the pro-inflammatory properties of processed food and underlying mechanisms such as gut microbiome modulation and augmented intestinal permeability driven by high sugar consumption (22). Alternatively, they might stem from excessive intake of empty calories resulting in a deficiency of essential nutrients required for optimal nervous system function (e.g., micronutrients, tryptophan, PUFAs). The Mediterranean diet has consistently showed promising effects in reducing anxiety, irrespective of the specific MD index used, in both sexes. However, the reported associations of specific Mediterranean food staples are inconsistent, and interpretation should be drawn cautiously due to differing methodologies in MD calculation. Notably, the MD aligns with the GDA, promotes anti-inflammatory foods, and recommends a diverse diet. The MD is also high in antioxidants and phytochemicals, and balanced in acid–base load, which have been associated with lower anxiety symptoms (7, 84, 88). These benefits might be ascribed to molecular cascades that reduce oxidative stress and its impact on cellular structures such as mitochondria and DNA, both influencing psychiatric disorders through mechanisms such as telomere shortening. Further, changes in synaptic oxidation and oxygen levels could cause neurotransmitter decline. Phytochemicals might also regulate antioxidant enzyme gene expression, aiding in oxidative stress reduction (183) and act as acid–base regulators (184), highlighting the interconnected roles of these dietary components in mental health. Other DPs, like the Nordic diet, and diets rich in HEI adequacy components, share nutritional profiles similar to the Mediterranean diet. Further research on DQIs associated to the MD, and common inherent properties of distinct dietary patterns, would be crucial for several reasons. First, the MD is geographically specific to the Mediterranean region, emphasizes local and seasonal foods which are more nutritious, and caters to diverse metabolic needs, including ethnicity-related microbiome variations for optimal nutrient absorption. Second, local consumption is environmentally sustainable, reducing emissions, pesticide use, and carbon footprint. Hence, the focus should shift from pushing toward the adoption of MD universally to tailoring diets based on its beneficial properties in accordance to local food supply. This approach aligns with findings that local food purchases correlate with higher diet quality, as per the AHEI-2010 (185), advocating for diet individualization to enhance health outcomes and sustainability. Regarding findings on plant-based foods, no association with anxiety occurs when fruits and vegetables are pooled together for either males, females, or mix samples, but when accounted for separately (14, 98, 120–124). The same has been found for gut microbiome when differentiating between fruits and vegetables HEI scores (33). Consistently, fruits and vegetables are characterized by different nutritional properties with vegetables presenting a higher Overall Nutritional Quality Index, although nutrient-specific content appears to be serving-dependent and fruits show double the antioxidant activity of vegetables (186). Future studies should then look at vegetables and fruits categories individually and, when possible, also distinguish food groups within categories (e.g., leafy greens, cruciferous, citrus, berries, etc.) which might still show unique nutritional profiles (186). A similar approach should be applied to vegetarian and vegan diets: pooling vegetarian and vegan diets should in fact be avoided given non-negligible dissimilarities between the consumed food groups. Also, most studies adopted self-reported questionnaires or relied on empirically derived DPs. The Plant-based Diet Index (PDI) exists and differentiates between healthy and unhealthy plant-based choices, offering a potential standard for evaluating dietary adherence. Adopting the PDI could thus enhance study comparability and reduce information bias by providing a nuanced approach to assessing plant-based diets. Given the advantages of *a priori* dietary indexes and the consistent effects of a processed diet on both anxiety and the gut microbiome, future studies should also focus on the validation of a Western Dietary Index.

**Table 1:** Summary of the most relevant included findings of dietary effects on anxiety (reported for adjusted models and odd ratios only).

	Reference	Design	*n*	Age range	Gender	Dietary variable	Outcome	Statistics	F/M	F	M
Dietary quality indices	Dehghan et al. (7)	Cross-sectional	350	14–16	F	DII – cat	DASS-21 – cat	LM	-	ns	-
	Salari-Moghaddam et al. (48)	Cross-sectional	3,363	18–55	F/M	DII – cat	HADS – cat	ORs	↑	ns	ns
	Salari-Moghaddam et al. (9)	Cross-sectional	3,363	18–55	F/M	FDII – cat	HADS – cat	ORs	↑	↑	ns
	Attlee et al. (47)	Cross-sectional	260	19–22	F	DII – cat	DASS-21 – cat	ORs	-	↑	-
						DII – con			-	↑	-
	Phillips et al. (46)	Cross-sectional	2047	50–69	F	E-DII – cat	HADS – cat	ORs	↑	↑	ns
	Varaee et al. (49)	Cross-sectional	5,579	20–70	F/M	DIS – cat	DASS-21 – cat	ORs	↑	-	-
	Isgin-Atici et al. (67)	Cross-sectional	272	13–18	F	HEI-2010 – cat	PMSS-anxiety	ANOVA	-	↓	-
	Christensen et al. (68)	Cross-sectional	77	18–25	F	HEI-2010 – con	APSQ – cat	Welch’s T-test	-	ns	-
	Taylor et al. (30)	Cross-sectional	133	25–45	F/M	HEI-2010 – con	DASS-42 – con	GLMM	ns	ns	ns
	Saneei et al. (70)	Cross-sectional	3,363	18–55	F/M	AHEI-2010 – cat	HADS – cat	ORs	↓	↓	ns
	Gibson-Smith et al. (69)	Cross-sectional	1,634	18–65	F/M	AHEI-2010 – cat	BAI – con	LM	↓	-	-
							CIDI – cat		ns	-	-
	Poorrezaeian et al. (76)	Cross-sectional	360	20–49	F	DDS – cat	DASS-21 – cat	ANOVA	-	↓	-
	Jiang et al. (77)	Longitudinal	924	18–45	F	DDS – cat	SAS – cat	LMM	-	↓	-
Dietary patterns	Sadeghi et al. (98)	Cross-sectional	3,172	18–55	F/M	MDScale – cat	HADS – cat	ORs	↓	-	-
	Gibson-Smith et al. (69)	Cross-sectional	1,634	18–65	F/M	MedDiet Score – cat	BAI – con	LM	↓	-	-
							CIDI – cat		ns	-	-
	Boaz et al. (14)	Cross-sectional	3,271	18+	F/M	MedDiet Score – cat	GAD-7 – con	rho	-	↓	↓
	Michalak et al. (103)	Cross-sectional	4,181	18–65	F/M	VEG – self-reported	CIDI – cat	ORs	↑	-	-
	Beezhold et al. (104)	Cross-sectional	620	25–60	F/M	VEG – self-reported	DASS-21 – con	ANOVA	ns	ns	↓
						VG – self-reported			↓	↓	↓
	Rossa-Roccor et al. (108)	Cross-sectional	339	-	F/M	Plant-based pattern	GAD-7 – con	LM	ns	-	-
				*young adults		Junk food pattern (WD)			↑	-	-
	Mousavi et al. (115)	Cross-sectional	3,362	18–55	F/M	PDI and hPDI – cat (VEG)	HADS – cat	ORs	↓	-	-
						uPDI – cat (WD)			↑	-	-
	Bakhtiyari et al. (109)	Cross-sectional	1,782	18–35	F/M	Processed food – cat (WD)	STAI-T – cat	ORs	↑	-	-
							STAI-S – cat		↑	-	-
	Hosseinzadeh et al. (114)	Cross-sectional	3,846	20–55	F/M	Fast food pattern (WD)	HADS – cat	ORs	ns	ns	ns
						Western pattern (WD)			ns	ns	ns
	Vilela et al. (110)	Longitudinal	185	20–40	F	Processed pattern (WD)	STAI-S – con	LMM	-	ns	-
	Yazdi et al. (111)	Cross-sectional	3,063	-	F/M	Western pattern (WD)	HADS – cat	ORs	↑	-	-
				*employees							
	Xu et al. (112)	Cross-sectional	1,360	45–59	F/M	Western pattern (WD)	GAD-7 – cat	ORs	↑	-	-
	Weng et al. (113)	Cross-sectional	5,003	11–16	F/M	Traditional pattern	SCARED – cat	ORs	ns	-	-
						Snack pattern (WD)			↑	-	-
						Animal food pattern			↑	-	-
	Salehi-Abargouei (116)	Cross-sectional	3,846	18–55	F/M	Omnivore pattern	HADS – cat	ORs	-	ns	ns
Foods	Tuck et al. (122)	Cross-sectional	428	18–60	F/M	FVI – con	HADS – con	LM	ns	-	-
						Savory snacks			↑	-	-
	Brookie et al. (124)	Cross-sectional	422	18–25	F/M	FVI – con	HADS – con	LM	ns	-	-
	Wattick et al. (123)	Cross-sectional	1,956	18+	F/M	FVI – con	Healthy Days (CDC) – cat	ORs	-	ns	ns
						Added sugar foods			-	↑	↑
	Saghafian et al. (120)	Cross-sectional	3,362	18–55	F/M	FI – cat	HADS – cat	ORs	-	↓	ns
						VI – cat			-	ns	↓
						FVI – cat			-	ns	ns
	Boaz et al. (14)	Cross-sectional	3,271	18+	F/M	VI – con	GAD-7 – con	rho	-	↓	ns
						FI – con			-	ns	ns
						Whole grains			-	↑	ns
						Processed meat			-	↑	↑
						Salty snacks			-	↑	↑
						Sweetened beverages			-	↑	↑
	Gibson-Smith et al. (121)	Cross-sectional	1,634	18–65	F/M	FI – con	BAI – con	LM	ns	-	-
						VI – con			↓	-	-
						Whole grains – con			↓	-	-
	Sadeghi et al. (98)	Cross-sectional	3,172	18–55	F/M	FI – con	HADS – cat	ORs	↓	-	-
						VI – con			↓	-	-
	Sadeghi et al. (100)	Cross-sectional	3,172	18–55	F/M	Whole grains – cat	HADS – cat	ORs	-	↓	ns
						Refined grains – cat			-	↑	ns
	Abbaszadeh et al. (92)	Cross-sectional	181	18–25	F	Whole grains	DASS-21 – con	LM	-	ns	-
	Yannakoulia et al. (129)	Cross-sectional	853	-	F/M	Read meat	STAI-S – con	LM	-	↑	ns
				*adults		Sweets			-	↑	-
	Darooghegi Mofrad et al. (131)	Cross-sectional	482	20–50	F	Red meat	DASS-21 – cat	ORs	-	↑	-
	Alharbi et al. (130)	Cross-sectional	92	19–45	F	Red meat	HADS – cat	ORs	-	↑	-
Nutrients	Hajihashemi et al. (140)	Cross-sectional	2,033	18+	F/M	CQI	HADS – cat	ORs	ns	-	-
						PQI			ns	-	-
						FQI			↓	-	-
	Daley et al. (151)	Cross-sectional	7,635	25–30	F	PUFA n-3 – con	self-reported	ORs	-	ns	-
						PUFA n-6 – con			-	↓	-
						PUFA n-9 – con			-	↑	-
	Kiecolt-Glaser et al. (153)	12 weeks RCT	68	21–29	F/M	PUFA n-3 *2.5 g EPA, 348 mg DHA	BAI – con	LMM	↓	-	-
	Watanabe et al. (150)	52 weeks RCT	80	20–59	F	PUFA n-3 *1.2 g EPA, 600 mg DHA	HADS – con	LMM	-	ns	-
	Wilson and Madrigal (152)	Cross-sectional	54	-	F	PUFA n-3 – con	BAI	rho	-	↓	-
				*college students		PUFA n-6 – con			-	ns	-
	Saghafian et al. (156)	Cross-sectional	3,362	18–55	F/M	Fibers – cat	HADS – cat	ORs	ns	↓	ns
	Johnstone et al. (29)	4 weeks RCT	64	18–25	F	7.5 g GOS	STAI-T – con	ANOVA	-	↓* > anx group	-
	Berding et al. (157)	4 weeks RCT	18	18–40	F	12.5 g PDX	HADS – con	ANOVA	-	ns	-
	Barfoot et al. (154)	2 weeks intervention	38	-	F	Increased flavonoid intake	STAI-S – con	LMM	-	↓	-
				*new mothers			STAI-T – con		-	ns	-
	Parilli-Moser et al. (135)	6 months RCT	63	18–33	F/M	Polyphenols from roasted peanuts	HADS – con	Wilcoxon’s test	↓	-	-
								rho	↓	-	-
	Lamport et al. (155)	12 weeks intervention	25	40–50	F	Polyphenols from grape juice	STAI-SF – con	ANOVA	-	ns	-

DII, Dietary Inflammatory Index; HEI, Healthy Eating Index; AHEI, Alternative HEI; DDS, Dietary Diversity Score; MDScale, Mediterranean Diet Scale; MedDiet Score, Mediterranean Diet Score; VEG, vegetarian; VG, vegan; WD, Western Diet; FVI, fruit-vegetable intake; FI, fruit intake; VI, vegetable intake; PUFA n-3, omega-3 polyunsaturated fatty acids; PUFA n-6, omega-6 polyunsaturated fatty acids; PUFA n-9, omega-9 polyunsaturated fatty acids; EPA, Eicosapentaenoic Acid; DHA, Docosahexaenoic Acid; GOS, galacto-oligosaccharides; PDX, polydextrose; DASS, Depression Anxiety Stress Scale; HADS, Hospitality Anxiety Depression Scale; BAI, Beck Anxiety Inventory; GAD-7, Generalized Anxiety Disorder-7 items; CIDI, composite international diagnostic interview; CDC, Center for Disease Control and Prevention; SCARED, Screen for Child Anxiety Related Disorders; cat, categorical; con, continuous; LM, linear regression models; LMM, linear mixed-effects models; ORs, odd ratios; rho, Pearson’s correlation coefficient; CQI, carbohydrates quality index; PQI, proteins quality index; FQI, fat quality index.

Consistent associations have been found between anxiety and gut microbiome genera across studies, i.e., increased *Bacteroides, Escherichia/Shigella, Tyzzerella, Collinsella*; decreased *Faecalibacterium, Roseburia, Subdoligranulum*, and genera from the Lachnospiraceae family. The same bacteria have also been linked to distinct dietary variables: higher HEI index and consumption of healthy dietary components are associated with lower abundance of *Bacteroides, Escherichia/Shigella, Tyzzerella, Collinsella* genera among others, and higher abundance of *Faecalibacterium* and *Subdoligranulum.* On the other hand, unhealthy Western diets and nutrients mostly present with associations in the opposite direction, i.e., loss of beneficial butyrate-producing bacteria, dominance of Bacteroidetes, and promotion of pro-inflammatory bacteria. Mixed results exist for fat intake and whether it is beneficial or detrimental to gut microbiome could depend on fat type and ratios rather than absolute values. Interestingly, most bacteria associated with diet and anxiety belong to the Firmicutes and Bacteroidetes phylum. As preclinical studies show specific associations of diet and microbiome with Firmicutes/Bacteroidetes ratio, the observed effects could depend on microbes’ inhibitory and excitatory interactions happening at the community-level (187), rather than fold differences at the genera and species level. This would also explain the conflicting results, and hint toward the benefit of network-based approaches. Further – when possible and depending on research aims – metagenomics, transcriptomics, and metabolomics information should also be integrated as ecological metabolic function could differ despite similar microbial structures (188). Recently, researchers have started considering microbiome-mediation models, supported by preliminary results (32, 35, 182). One’s temporal microbiome would affect one’s individual responsiveness to diet and anxiety risk and could then be responsible for distinct levels of susceptibility to anxiety and strength of associations with microbiome and short-term dietary intake. This concept aligns with a recent review from Ortega et al. (189) which emphasizes the impact of gut microbiota on neuromodulation, endocrine functions, and immune responses in bipolar disorder, a mood disorder which can be accompanied by anxiety. Building on these pieces of evidence, we suggest that a research shift toward gut microbiome-mediated mechanisms would benefit interventional designs and offer personalized translational opportunities resulting in more efficacious management of anxiety symptomatology. We also want to draw attention to the importance of assuming an integrative perspective that takes into account the interconnectedness of multiple body’s systems, e.g., inflammatory, metabolic, nervous systems, analogously to what recently proposed for the clinical management of spinal cord injury patients (190). All these systems contribute to anxiety pathophysiology and should thus be accounted for when developing clinical treatments and during patients’ evaluation.

**Figure 5:**
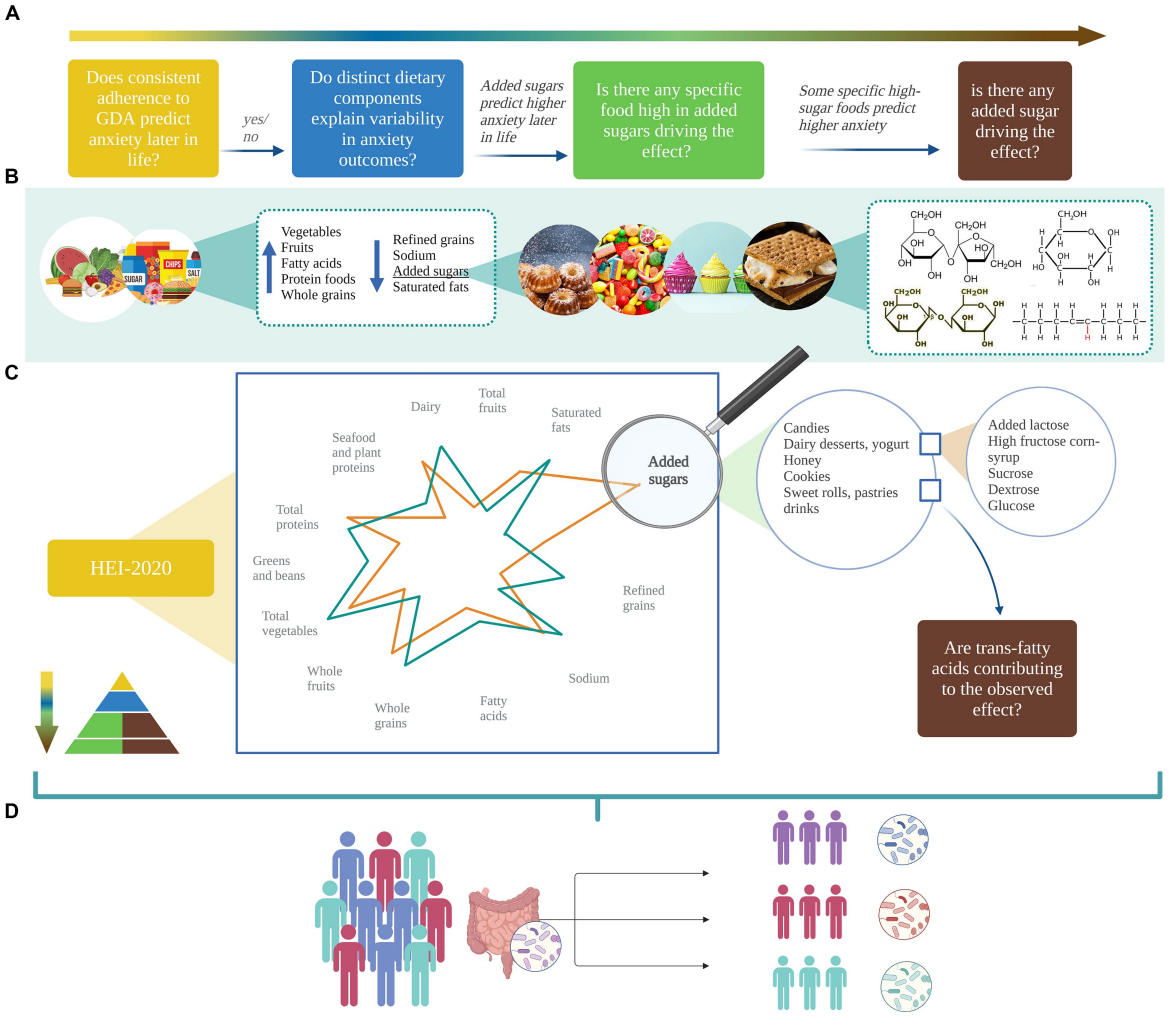
Example of the proposed method: a multilevel approach for dietary analysis should be adopted based on **(A)** the main research questions, **(B)** the hierarchical diet composition, and **(C)** guided by empirical results. Here, the analysis pipeline starts from the main independent variable of interest – the HEI-2020 – and moves down to added sugars based on DPs analysis and/or, e.g., existing evidence of sugars effects on anxiety. Differences in consumption of sweetened foods and beverages are then compared and driving effects of specific added sugars (e.g., lactose, corn-syrup, sucrose) explored. Although multilevel analyses of fats are not initially performed, downstream results (e.g., differences in consumption of ultra-processed food such as cookies and pastries) prompt exploratory analysis of trans-fatty acids. Note that the pipeline can flow in each direction, start at any hierarchical level, and include variable number of layers based on (i) main objective, (ii) relevant literature, (iii) results, (iv) staff and research resources. However, the rationale behind authors’ choices should always be transparent and outlined. As shown in **(D)**, gut microbiome should be measured and included as a stratification or moderation factor. Created with and adapted from BioRender.com.

Notably, the present study suggests sex-specific other than population-specific effects of both gut microbiome and diet on anxiety. Indeed, diet and gut microbes might affect non-anxious, sub-clinically, and clinically anxious individuals heterogeneously as also suggested by our previous work (191). This can be seen in (i) Gibson-Smith et al. (69) study where the HEI and the MedDiet Score predicted anxiety severity but not diagnosis, (ii) Johnstone et al. (29) study where only highly anxious females benefitted from GOS supplementations, and in (iii) contradictory findings across gut microbiome studies looking at non-anxious, subclinical, and clinical populations. Based on the sample of interest, distinct anxiety scales should be chosen as more sensitive to symptoms. For example, the STAI and DASS scales might be preferrable when including subclinical samples, the Hospitality Anxiety Depression Scale (HADS) would be best used with medical patients, whereas the BAI would better distinguish anxiety from depression (192). Other considerations include ambiguities arising from categorization of both anxiety and dietary variables that sometimes was arbitrary, sometimes data-driven, and most of the times prevented comparison between studies and hindered results interpretability. On the light of it, categories should be adopted only (i) with a clear rational, (ii) when consensus on cut-off points exists, (iii) when the distribution of the data justifies so. Alternatively, *a priori* grade system might aid subject stratifications at recruitment and/or study enrolment depending on the specific research question. Data should then be analyzed categorically only if statistical pair-wise comparison justifies so and if there’s no reason to believe that risk varies between categories (51). Categorical versus continuous methods could also be compared against each other and both results included for publication. Finally, we suggest that future research in the field of nutrition should try to adopt a funnel-shaped method (Figure 5) flowing upwards and downwards based on the specific research question, prioritizing *a priori* dietary indexes when available, and adopting data-driven methods when samples are large enough, for explorative purposes or to periodically validate existing guidelines and update theoretical knowledge. To optimize such a multilevel approach and integrate distinct levels of information, different nutritional assessment methods (e.g., food recordings, food-frequency questionnaires) should be combined and tailored to the sample of interest. Further, to elucidate the mediator and moderator roles proposed for defined bacterial genera and to identify additional genera/species, future studies should focus on assessing changes in the gut microbiome and establishing baseline profiles during interventional research. In scenarios where interventional studies are impractical, effort should be directed toward identifying temporal and dynamic microbiome features across multiple time points in observational and epidemiological studies. As done by Rossa-Roccor et al. (108), research studies should be contextualized on a biopsychosocial framework that accounts for the multifactorial nature of the relationship between diet, anxiety, the gut microbiome, and the existing confounding variables.

In summary, the present study depicts the current state of nutritional research on anxiety while pinpointing methodological gaps that need further improvement and highlighting the importance of integrating the gut microbiome. By integrating available evidence, some bacterial genera that likely mediate the relationship between diet and anxiety can be identified (Figure 4A). Existing translational opportunities and knowledge gaps that need further research are also highlighted with the expectation that professionals and practitioners will yield valuable insight and future studies will provide the requisite evidence. Indeed, there is good evidence for interplays between diet, the gut microbiome and anxiety that could be translated into the clinical and community settings. Probiotics and prebiotics have shown potential and might work best when combined and along complementary dietary recommendations. Most importantly, prebiotics have shown preliminary results in affecting both anxiety symptoms and emotional eating. Although the underlying mechanisms and directionality of effects are not clear, this paves the way for new opportunities to break the anxiety-eating cycle upon further investigations on preclinical and human models.

## Data availability statement

The original contributions presented in the study are included in the article/supplementary material, further inquiries can be directed to the corresponding author.

## Author contributions

MeB: Writing – review & editing, Writing – original draft, Investigation, Conceptualization. IZ: Writing – review & editing, Writing – original draft, Investigation. NJ: Writing – review & editing. MaB: Writing – review & editing, Writing – original draft, Supervision, Investigation, Funding acquisition, Conceptualization. KC: Writing – review & editing, Supervision, Funding acquisition, Conceptualization.
